# From Trust in Automation to Trust in AI in Healthcare: A 30-Year Longitudinal Review and an Interdisciplinary Framework

**DOI:** 10.3390/bioengineering12101070

**Published:** 2025-10-01

**Authors:** Kelvin K. L. Wong, Yong Han, Yifeng Cai, Wumin Ouyang, Hemin Du, Chao Liu

**Affiliations:** 1 AI Creativity Laboratory, Academy of Fine Arts, Hunan Normal University, Changsha 410081, China; kelvin.wong@ieee.org; 2 Department of Mechanical Engineering, Division of Biomedical Engineering, University of Saskatchewan, Saskatoon, SK S7N 549, Canada; 3 Faculty of Innovation and Design, City University of Macau, Macau 999078, China; u23092110161@cityu.edu.mo (Y.H.);; 4 School of Innovation and Design, Shenzhen Technology University, Shenzhen 518000, China; 5 Graduate School of Global Convergence, Kangwon National University, Chuncheon 24341, Republic of Korea; 6 Yangtze Delta Region Institute, Tsinghua University, Jiaxing 314006, China

**Keywords:** human-AI in healthcare, XAI in healthcare, I-HATR framework, XAI-HCI alignment, human-centered and trustworthy AI

## Abstract

Human–machine trust has shifted over the past three decades from trust in automation to trust in AI, while research paradigms, disciplines, and problem spaces have expanded. Centered on AI in healthcare, this narrative review offers a longitudinal synthesis that traces and compares phase-specific changes in theory and method, providing design guidance for human-AI systems at different stages of maturity. From a cross-disciplinary view, we introduce an Interdisciplinary Human-AI Trust Research (I-HATR) framework that aligns explainable AI (XAI) with human–computer interaction/human factors engineering (HCI/HFE). We distill three core categories of determinants of human-AI trust in healthcare, user characteristics, AI system attributes, and contextual factors, and summarize the main measurement families and their evolution from self-report to behavioral and psychophysiological approaches, with growing use of multimodal and dynamic evaluation. Finally, we outline key trends, opportunities, and practical challenges to support the development of human-centered, trustworthy AI in healthcare, emphasizing the need to bridge actual trustworthiness and perceived trust through shared metrics, uncertainty communication, and trust calibration.

## 1. Introduction

As a rapidly diffusing technology, artificial intelligence (AI) is now embedded across industries and daily life [1]. Beyond being one of the most closely watched trends in research and practice, AI is projected to add roughly 13 trillion USD to the global economy by 2030 [2]. Thanks to its capacity to process and analyze large-scale data quickly and accurately, AI has shown particular promise in high-stakes decision domains such as healthcare [3], aviation [4], defense [5], finance [6], and law [7]. In healthcare, AI is driving profound change [8], demonstrating substantial potential in areas including electronic health record mining [9], medical image diagnosis [10], treatment planning [11], and clinical data interpretation [12].

Despite this momentum, AI brings material risks and uncertainties alongside new opportunities. Deep learning (DL) models already support high-risk tasks such as cancer diagnosis by extracting features from medical images to assist pathologists [13]. Yet the black-box nature of many models introduces opacity, limited interpretability, and potential bias, all of which can jeopardize clinical decision making [14,15]. Even in lower-risk consumer applications (e.g., entertainment or shopping), prior studies show that opacity diminishes understanding and precipitates trust failures. Parasuraman and Riley (1997) [16] characterized the downstream patterns as use, misuse, disuse, and abuse; Mehrotra et al. (2024) [17] provided a recent systematic review cataloging design levers for cultivating appropriate trust in human–AI interaction. Complementing these, Castelvecchi (2016) [18] emphasized the “black-box” nature of modern AI, and Retzlaff et al. (2024) [19] examined the hidden decision pathways between inputs and outputs—i.e., the lack of an intuitive mapping—contrasted post hoc and ante hoc approaches, and offered XAI design guidelines.

Trust itself is a core social construct that shapes interaction [20]. Historically, accuracy was typically prioritized over interpretability; for instance, Nussberger et al. (2022) [21] find that, although the public values interpretability, people often still prioritize accuracy. In response, recent scholarship has advanced explainable/transparent AI and, in high-stakes settings, arguments for using inherently interpretable models (e.g., Rudin, 2019 [22]), alongside improved uncertainty communication [19,21]. However, many methods introduce their own biases, rely on abstract visualizations or statistical surrogates, and may add complexity rather than reduce it [23]. More importantly, these technical paths often lack concrete guidance for incorporating human–computer interaction and human factors to improve real-world performance and adoption [19]. As AI diffuses, the challenges it raises exceed those of earlier technologies and demand cross-disciplinary responses [24,25]. Yet the XAI community in computer science and the human factors community in HCI often advance on separate tracks [19,26]. Put differently, XAI work is frequently optimized for algorithmic accuracy with limited attention to user-centered psychology, ergonomics, and design cognition, which leads to poor usability and explainability, low transparency, and inappropriate trust [19]. Many explainability efforts also underuse insights from non-AI disciplines on explanation, understanding, and trust [27,28]. Prior commentary warns that emphasizing the benefits of explaining black-box models can obscure important downsides. The field is therefore shifting from performance-centric AI toward user-centric AI [20], a pivot that is especially urgent in high-risk domains such as healthcare.

Trust is one of the most decisive elements in social interaction [29]. Over the past three decades, the notion of “human–machine trust” has likewise played a central role in the development and use of interactive systems [30]. Whether users can place appropriate trust in these systems often determines success or failure. Notably, maximizing trust is not always beneficial; over-trust can lead to harm and even fatal outcomes. In the 1990s, research focused on operators’ trust in automated systems, emphasizing reliability and predictability. By contrast, contemporary AI introduces data-driven learning, autonomy, and adaptation—capabilities that earlier technologies lacked. The rapid rise of deep learning and generative AI has brought new debates about explainability, fairness, and trustworthiness, prompting a fundamental rethinking of human–AI trust.

Although several high-quality review articles have examined trust in automation and AI [31,32,33,34,35], and a smaller number have focused specifically on trust in medical AI [36,37,38], three critical gaps remain. (1) There is still no longitudinal integration that traces the thirty-year evolution from “trust in automation” to “trust in AI,” which makes it difficult to reveal phase-specific differences in theories and methods. (2) The XAI research community and the HFE/HCI trust research community have long operated in parallel without convergence [20]. Research in computing and XAI focuses on actual trustworthiness, primarily driven by model performance and governance indicators. In contrast, research emphasizing human factors and human–computer interaction focuses on perceived trustworthiness, shaped by explanation quality and interaction experience. A deeper challenge lies in the fact that actual trustworthiness is not equivalent to perceived trustworthiness. In the literature, the transition from automation trust to AI trust has further entrenched this divide, as the two dimensions continue to be assessed within separate disciplinary frameworks. As a result, their evaluation criteria and reported outcomes are difficult to align within the same task unit, and a unifying framework that bridges actual and perceived trustworthiness is still lacking. (3) In healthcare contexts, research on trust calibration tends to focus on a single dimension (such as system performance), without systematically integrating user characteristics, contextual risk, and other interacting factors.

To address these gaps and highlight an interdisciplinary view of appropriate human–AI trust, especially in healthcare, we pose three research questions:

- From automation trust to AI trust, how has human–machine trust evolved over the past thirty years? This includes: A. What is the historical background of interpersonal trust and the early progress on trust in automation? B. How has human–AI trust evolved since the advent of AI systems?
- Through which key mechanisms do XAI and HCI, respectively, influence actual trustworthiness and perceived trustworthiness, and how can these lines of work be integrated into a unified framework that bridges the two?
- What research gaps, major challenges, and future opportunities define the current landscape of AI trust?

We aim to provide a foundation for cross-disciplinary research and to help designers in high-risk domains such as healthcare optimize user trust strategies in increasingly complex technical environments. To that end, we conduct a narrative review with critical synthesis.

Our contributions are threefold:

- We synthesize a thirty-year longitudinal evolution from “automation” to “AI”, summarizing phase-specific technical contexts, trust mechanisms, theoretical iteration, and practical foci.
- We compare trust research across the XAI research community and HCI and develop an Interdisciplinary Human-AI Trust Research (I-HATR) framework that places actual trustworthiness and perceived trustworthiness within the same coordinate system, providing a multidimensional understanding of human-AI trust and helping to bridge the gap between them.
- We outline future research opportunities, major challenges, and actionable directions based on current trends

## 2. Methods

### 2.1. Scope and Positioning

We conduct a narrative review with critical synthesis along two axes:

(1) A longitudinal account of the shift from trust in automation to trust in AI.

(2) A cross-disciplinary alignment of XAI and HCI research paths.

In what follows, Section 3 addresses RQ1 by tracing phase-specific changes from automation to AI; Section 4 addresses RQ2 by aligning XAI and HCI/HFE within I-HATR on the same task unit; and Section 6 addresses RQ3 by distilling gaps, limitations, and future directions, including task-level joint reporting and calibration guidance.

Our aim is to map the landscape, compare paradigms, and propose a research agenda rather than to exhaust all studies or perform a meta-analysis. We include English, peer-reviewed publications from 1995 through June 2025. Eligible types are journal and conference papers spanning human–computer interaction (HCI), human factors engineering (HFE), cognitive psychology, computer science, and AI (with emphasis on XAI). We focus on HCI, HFE, and cognitive psychology on the human side and computer science and AI (with emphasis on XAI) on the system side because this set maps onto a trustor–trustee–context triad and covers the main measurement families (self-report, behavioral, psychophysiological) alongside technical metrics (calibration, fidelity and stability, uncertainty). This selection enables alignment and joint reporting within I-HATR at the same task unit. We focus on high-risk applications, especially healthcare.

Three recent surveys provide context for our positioning. Glikson & Woolley (2020) review empirical research on human trust in AI across domains, with broad coverage but limited emphasis on healthcare-specific operationalization [34]. Asan et al. (2020) focus on clinicians’ trust and adoption issues in medical AI, offering domain insights but with less integration to XAI evaluation metrics or task-level reporting [36]. Catapan et al. (2025) synthesize trust in digital healthcare among consumers/patients and healthcare professionals, highlighting determinants and outcomes rather than cross-walking human-factors measures with technical indicators [38]. In contrast, our review (i) traces a 30-year evolution from automation to AI; (ii) introduces I-HATR to align actual and perceived trustworthiness at the same task unit; and (iii) operationalizes this alignment via a task-oriented XAI taxonomy and joint reporting of calibration/uncertainty with trust/behavioral/cognitive measures to support study design and cross-disciplinary comparability. We further outline implementation anchors for compliance and societal impact in high-risk healthcare, aiming to bridge lab-to-clinic gaps.

### 2.2. Search and Selection

We ran reproducible topic/abstract/keyword searches in Web of Science, Scopus, ACM Digital Library, and PubMed and used Google Scholar for forward/backward snowballing and gray-literature leads (only English, peer-reviewed sources were retained). Records were exported to Zotero for de-duplication and screening.

Keywords and search strategy.

We combined four concept blocks consistently across databases:

Trust (e.g., trust, appropriate trust, reliance, trust calibration); System/XAI (e.g., explainable AI, interpretability, transparency, uncertainty; common methods such as counterfactuals, SHAP, LIME, saliency maps); HCI/HFE (e.g., human–computer interaction, human factors, mental models, cognitive load, NASA-TLX, psychophysiology); Healthcare domain (e.g., health/medical/clinical context, patients, hospitals, diagnosis, triage, including CDSS).

For each database we adjusted only field tags (e.g., MeSH vs. title/abstract vs. index terms) while keeping the same block combination. Last search: June 2025. Representative term lists and minor phrasing variants are provided in Supplementary Table S1.

Inclusion criteria: studies directly addressing the concepts, models, measures, or empirical evidence of human–machine or human-AI trust, and involving at least one AI-era element (e.g., explainability, calibration/fidelity, uncertainty communication, reliance/disuse). Title/abstract screening and full-text review were conducted by the first author; a second author audited a sample of records and discussed discrepancies to improve consistency. Decisions were logged to enable retracing. We also reviewed selected classic and recent survey papers.

Framework synthesis.

In parallel with evidence collection, we organized the findings into an interdisciplinary crosswalk (I-HATR) that aligns system-side actual trustworthiness with human-side perceived trustworthiness on the same task unit. We first defined the task and risk class, then coded each included study for determinants (user, system, context), measures (technical: calibration, fidelity/stability, uncertainty, robustness; human: self-report, behavioral, psychophysiological), and key contextual variables (e.g., time pressure and oversight). We mapped metrics and measures to the two axes and identified potential calibration loops such as uncertainty communication and escalation/oversight. From this mapping we derived a minimal set of joint reporting items so that calibration/uncertainty can be co-reported with trust, reliance, and cognitive measures for the same task. Coding disagreements were audited by a second author and resolved by discussion. The framework is intended as design scaffolding rather than a prescriptive checklist, and no numerical aggregation was attempted.

## 3. Longitudinal Evolution: From Trust in Automation to Trust in AI

### 3.1. Foundations of Trust in Automation

Across history and cultures, the importance of interpersonal trust has been repeatedly emphasized, offering insight into what constitutes appropriate trust in modern human–machine collaboration [29]. Early work on the precursor of human–machine trust—interpersonal trust—framed trust as expectation or confidence in another party based on reliance on cooperative or beneficial behavior (e.g., Rotter, 1967 [39]; Muir, 1987 [40]; Hwang and Buergers, 1997 [41]). Trust has also been treated as both a relatively stable attitude or trait and a situational state that varies with context [42].

Bainbridge’s “Ironies of Automation” highlighted that system understandability is a prerequisite for operator trust; more broadly, it described a mismatch between human expectations and the actual capabilities of automated systems, an irony whereby automation intended to increase efficiency can complicate human–machine cooperation when trust misaligns [43]. Although Bainbridge did not use the term “trust in automation,” the work implicitly addressed operator attitudes toward and changes in trust. The first study explicitly centered on trust in automation emerged in 1987, when Muir proposed a dynamic model of human–automation trust for calibrating users’ reliance on decision aids [40].

From 1987 to the mid-1990s, annual publication counts on human–automation trust remained low, with scattered contributions. Even so, these formative efforts established trust as a key variable and underscored the decisive role of system understandability in cooperation.

### 3.2. Trust in Automation

#### 3.2.1. Technological Trajectory

Automation refers to the full or partial substitution of functions previously performed by human operators [44]. In healthcare during the same period, clinical decision support systems (CDSS) were largely rule- or knowledge-based (e.g., drug–drug interaction checks, dosing aids, threshold-based alerts), with relatively narrow functionality and an emphasis on stability and usability [45]. For example, early prescription review and medication alerting could automatically intercept contraindicated interactions, yet they relied on fixed rules and thresholds, lacked adaptation and explicit uncertainty, and often required human adjudication under complex or unanticipated inputs [45]. Hoff and Bashir (2015) identified system failure rate (performance) and feedback transparency as core determinants of trust [32]. In clinical settings, the analog of “state displays” includes clear presentation of risk scores, confidence intervals, intended populations, and data provenance [46]. Trust has been recognized as a central variable explaining both resistance to use (under-trust) and over-reliance (over-trust) on automated systems [47]. As a high-risk, heavily regulated domain, the success of medical AI deployment depends heavily on frontline clinicians’ trust in system outputs [48].

Under-trust. High-frequency, low-specificity alerts in practice can induce alert fatigue and low trust; many medication safety warnings are overridden and have been linked to elevated risks of prescribing errors or adverse events [49,50].

Over-trust. Over-reliance on radiology CAD can degrade reading quality or introduce bias; software failures in medical devices have also caused severe harm when operators relied too heavily on automation (e.g., Therac-25), underscoring lethal risks when independent verification and uncertainty cues are absent [51,52].

**Figure 1:**
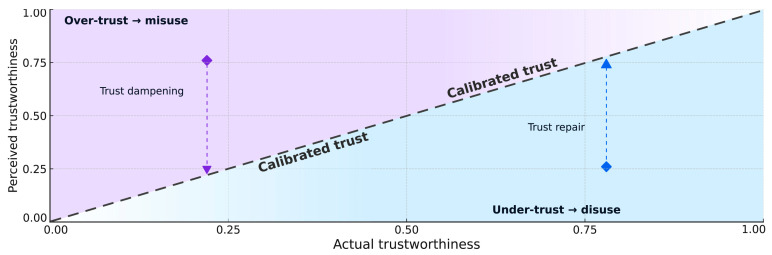
Conceptual schematic of trust calibration in human–machine systems. Axes are normalized (0–1); dashed line y = x denotes calibrated trust; upper region = over-trust/misuse, lower region = under-trust/disuse; vertical moves illustrate perception-only adjustments.

Figure 1 visualizes trust calibration on a normalized 0–1 scale. The *x*-axis denotes actual trustworthiness (system competence and governance), and the *y*-axis denotes perceived trustworthiness (users’ belief and reliance). The dashed diagonal (y = x) marks calibrated trust; points above the line indicate over-trust with risk of misuse, whereas points below indicate under-trust with risk of disuse [36]. In medical AI, when perceived trust exceeds actual trustworthiness, users enter the over-trust region, which can lead to misuse and patient harm [16,44,51]. Conversely, when perceived trust falls below actual trustworthiness, under-trust may occur—e.g., clinicians underestimate system capability due to prior negative experiences or opaque uncertainty, resulting in poorer monitoring efficiency, workload imbalance, and eventual abandonment or avoidance [53].

#### 3.2.2. Theory and Paradigm Shifts

Trust is a cross-disciplinary topic spanning psychology, sociology, and economics. Castaldo [54] applies a content meta-analysis across these fields, compiling a corpus of over 300 definitional formulations and grouping them by recurring semantic elements; seventy-two representative definitions are tabulated as the basis for subsequent comparison and synthesis. This heterogeneity makes it difficult to build a unified research program on human–machine trust atop prior work [32,55]. Accordingly, multiple qualitative stances coexist: trust as willingness [56], belief/attitude [31], affective response [57], mutual understanding [58], or reliance behavior [59].

Among these theories, the organizational trust model of Mayer, Davis, and Schoorman (1995) is particularly influential [56]. It distinguishes trust from its antecedents and outcomes (i.e., risk-taking in relationships) [32,60] and has become a prominent theoretical basis for trust in automation and AI [34,61]. The model posits that trust arises under risk and vulnerability; without risk, the situation reflects confidence rather than trust. Trust is defined as the willingness of a party to be vulnerable to another’s actions, based on the expectation that the other will perform actions important to the trustor, irrespective of the trustor’s ability to monitor or control the other [56]. Building on this, we operationalize trust in intelligent medical systems as follows: in uncertain and potentially risky clinical contexts, an individual clinician (e.g., a pathologist) is willing to adopt and rely on system recommendations—despite limited ability to monitor or intervene—based on expectations about the system’s ability, integrity, and benevolence, in order to achieve specific clinical goals (e.g., tumor diagnosis).

**Figure 2:**
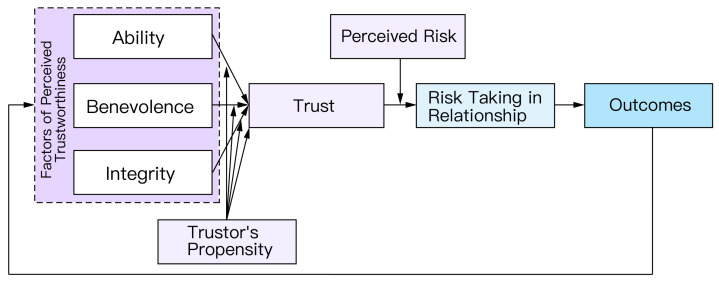
Mayer, Davis, and Schoorman’s model of trust decision making [56].

Conceptually, judgments of perceived trustworthiness should be separated from the subsequent trust decision. As shown in Figure 2, perceived trustworthiness is formed from three attributes: Ability (can the system accomplish the task), Benevolence (does it advance the user’s or patient’s interests), and Integrity (does it adhere to rules, ethics, and agreements). The model makes explicit where trust “comes from” and “what it leads to”: trust is shaped jointly by these trustee attributes, the trustor’s propensity to trust, and risk appraisal; it then drives reliance behavior and interaction outcomes, which in turn update perceived trustworthiness.

Because interpersonal trust and trust in automation differ in important ways [60], the organizational framework by Mayer et al. does not map one-to-one onto automation contexts [56]. Lee and See [31] contextualized it by summarizing three bases of trust in automation—Performance, Process, and Purpose—which align with Ability, Integrity, and Benevolence, respectively. In healthcare: Performance concerns whether a device or system can competently support clinical tasks, reflected in diagnostic/triage accuracy, robustness, and reliability; Process concerns whether the system operates according to established procedures and safeguards; Purpose concerns whether the system prioritizes patient and team interests, for example, by balancing risks and benefits, honestly communicating uncertainty, and reducing workload.

The trust-in-automation paradigm is best characterized as system-centered empirical research [31]. Grounded in interpersonal trust theories, it emphasizes how system performance (capability, predictability, reliability) affects trust. Methodologically, this work relies on laboratory experiments and surveys that manipulate failure rates and feedback transparency; representative tools include the Trust in Automation Scale by Jian et al. (2000) [62]. Behavioral indicators (e.g., intervention frequency) are commonly used, supplemented by limited physiological measures such as eye-tracking. Overall, this phase shows a “mechanistic” orientation that treats human–automation trust as near-linear adjustment to input–output feedback and prioritizes quantitative indicators over psychological mechanisms [32]. It also centers on the system while giving less attention to individual differences and context. This approach suits rule-based automation but is less adequate for data-driven, learning, and adaptive AI, setting the stage for a subsequent shift toward trust in AI.

#### 3.2.3. Applications and Extensions

Parasuraman et al. (2000) reported an inverted-U relationship between trust and level of automation [63]. In clinical settings, this often implies that moderate automation—for example, “recommend/approve” clinical decision support systems (CDSS) or semi-automated devices with adjustable autonomy—more readily supports appropriate reliance, whereas fully autonomous, unattended closed-loop decision and actuation can provoke distrust or outright rejection [45,64]. Beyond healthcare, aviation, nuclear power, defense, and industrial control show similar patterns: higher reliability and clearer state visibility tend to increase trust, while alerts and interface cues are needed to calibrate both over- and under-reliance [16,31,65].

Within healthcare, work on trust in automation has focused on rule-based CDSS, alarm management, and imaging CAD. Early knowledge-base/threshold CDSS produced frequent, low-specificity prompts, contributing to alert fatigue, overrides, and eventual abandonment, which in turn erode trust [49]. Regarding explanation and uncertainty communication, multiple studies indicate that visual explanations alone do not necessarily improve appropriate trust; explanations must be task-aligned and accompanied by uncertainty and intended-use boundaries to enable informed uptake and avoid overtrust [66,67]. Overall, clinical scenarios corroborate the core propositions of the automation-trust era: reliability, transparency, and trust calibration are essential, but they must be evaluated and validated within concrete clinical tasks and workflows [45].

### 3.3. Trust in AI

**Figure 3:**
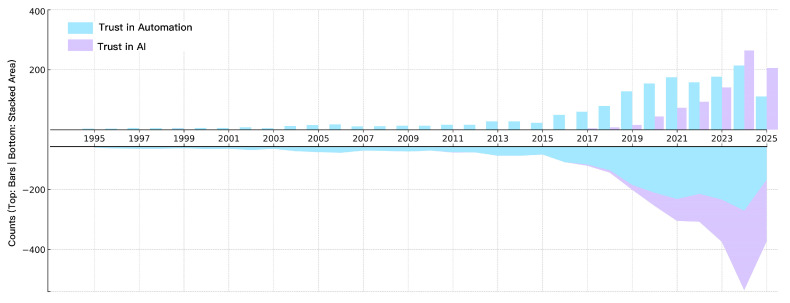
Trust in automation vs. trust in AI (1995–2025): annual comparison. Top panel—grouped bar chart; bottom panel—mirrored stacked area; both share the same yearly timeline. Last update: June 2025. The lower area is plotted as a negative mirror for visual balance; all counts are non-negative.

Drawing on nearly three decades of records, we observe a marked shift in emphasis from human–automation trust to human–AI trust (Figure 3). From 1995 to 2015, the literature was dominated by automation-oriented trust. After 2017, AI-focused trust rose rapidly and, in 2024, surpassed automation for the first time (264 vs. 214; 55.2%). As of the 2025 search cut-off, the gap widened further (206 vs. 111; 65.0%). Building on this shift, the following subsections discuss technological developments, theoretical and paradigm changes, and application extensions, and briefly point to the prospect of human–AI mutual trust.

#### 3.3.1. Technological Developments

Across history, major technological waves have reshaped both who we trust and how we evaluate trust. Automation trust is the precursor to AI trust, yet the two are often conflated in HMI/HCI scholarship [34]. The distinction matters: traditional automation can be exhaustively rule-based, with trust grounded in predictability and stability [16]. In contrast, AI is data-driven and adaptive; it updates its behavior through learning and can anticipate user needs [33,68,69]. Put simply, AI can learn and sometimes act in unexpected, hard-to-interpret ways [70].

This adaptivity brings flexibility but also new trust challenges: AI decision processes are frequently perceived as a black box, limiting transparency [22,71]. Trust, therefore, hinges not only on reliability but also on how users perceive and understand complex decision pipelines. XAI emerged to make these processes more transparent and, in turn, to foster trust [72]. Still, transparency does not eliminate irreducible uncertainty. The very complexity that enables rapid, context-sensitive responses also makes AI systems less stable and harder to predict than traditional automation [35]. As a result, cultivating trust in AI cannot rely on predictability alone.

A crucial implication is that trust and trustworthiness are not identical. One does not guarantee the other: people may trust an untrustworthy model and withhold trust from a highly trustworthy one [73]. In healthcare, a sophisticated classifier might accurately estimate cardiovascular risk from genetic, lifestyle, and metabolic features, yet clinicians may still withhold trust if the rationale is opaque. Conversely, even poorly performing models can attract trust simply because they present a persuasive GUI [74].

#### 3.3.2. Theory and Paradigm Shifts

Advances in AI inevitably reshape the structure of trust and challenge interpersonal trust concepts imported into human–AI settings [75]. People often apply social cognition to AI, treating it less like a tool and more like a potentially trustworthy social entity; boundaries between human–human and human–AI interaction are increasingly blurred. It also remains ambiguous whether trust judgments target the system, the developer/organization, or both [76]. Against this backdrop, human–AI trust becomes pivotal for uptake and effective use under uncertainty and complexity. Building on classic models (Mayer et al., 1995 [56]; Lee & See, 2004 [31]), Hoff & Bashir (2015) emphasize that trust governs willingness to rely on automation under risk [32]. In clinical AI, trust is similarly framed as stakeholders’ attitudes and reliance dispositions toward a system, providing a conceptual basis for designing decision support in complex settings.

Methodologically, the automation-trust tradition validated trust mechanisms through user experience and feedback in controlled studies [31]. For AI, evaluating trust also requires understanding internal mechanisms and users’ mental models of complex decisions [72]. Reflecting this, human–AI trust research in healthcare increasingly integrates machine learning perspectives, ethical responsibility allocation, and decision/cognitive engineering approaches [77,78].

#### 3.3.3. Application Extensions

The shift from rule-based to adaptive systems is well illustrated by recommender engines, voice assistants, and the early stages of autonomous driving, where outputs are continuously tailored to user behavior [71]. For instance, Netflix recommendations are individualized by mining viewing histories. This adaptivity pushes trust research beyond raw reliability toward interpreting system intent and boundaries of appropriate use [34].

Medical imaging is widely considered a high-potential beneficiary of AI [79]. Yet deployment remains uneven due to limited trust among clinicians, other stakeholders, and patients, as well as regulatory, forensic, and ethical constraints [80]. Similar dynamics—risk aversion and trust shortfalls—constrain adoption in other domains such as autonomous vehicles [81,82], intelligent assistants [83], and finance [84,85].

The phase-specific findings above provide the empirical basis for integrating actual (system-side) and perceived (human-side) trustworthiness. We carry these elements forward into I-HATR, where technical metrics (calibration, fidelity/stability, uncertainty, robustness) are paired with human-factors measures (validated trust scales, reliance/override behavior, cognitive load/psychophysiology) on the same task unit.

## 4. An Interdisciplinary XAI–HCI Framework for Human–AI Trust

Trust in AI is not merely an ethical add-on; it spans model performance, transparency, and explainability [29]. Within XAI, an explicit or implicit explainability–trust hypothesis assumes that explanations can promote or increase trust [86]. Indeed, building trust is often cited as a primary aim of explainability [87,88]. Recent debate on AI trustworthiness ranges widely—from how to help people trust AI to why some AI systems should not be trusted [19].

Evidence shows that most XAI methods are developed and assessed via computational evaluations, while potentially valuable human-centered evaluations (from HCI and HFE) are often underused [89,90]; only about 5% of explainability studies include a human-centered evaluation [91]. In practice, computational assessments prioritize actual trustworthiness because they support method development and debugging [64,92]. Yet actual trustworthiness shapes but does not determine users’ perceived trust [93]. HCI therefore focuses on perceived trust and the factors that influence reliance in context [48].

Our goal is not to rank these traditions but to align them. We propose the Interdisciplinary Human–AI Trust Research Framework (I-HATR) to bridge XAI and HCI, combining model-side trustworthiness with user-side perceived trust, especially for clinical AI. The framework is grounded in interdisciplinary process models [94] and responds to calls for user-centered, cross-disciplinary approaches [20]. Although motivated by healthcare, it generalizes to other high-stakes domains.

**Figure 4:**
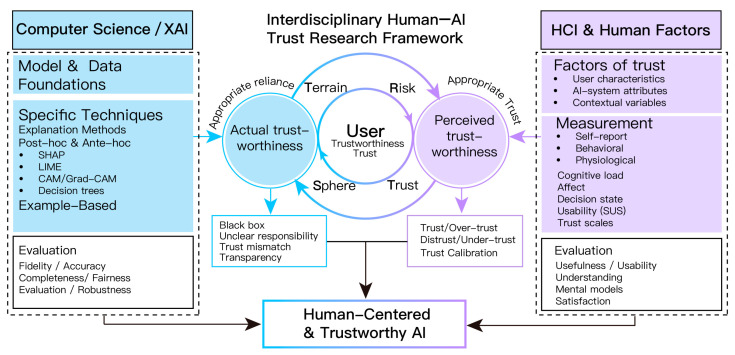
Interdisciplinary Human–AI Trust Research Framework (I-HATR). Hub-and-two-wings structure. The central hub is user-centered and includes five elements—trustworthiness, risk, user, sphere (individual–organization–community), and terrain (context) [20]. Left wing (XAI/computing): model, data, and explanation methods (e.g., SHAP, LIME, counterfactuals, saliency maps) plus evaluations of robustness/fairness/calibration to build actual trustworthiness; explanations and experiments can then shape perceived trust. Right wing (HCI/HFE): measures of cognitive load, affective experience, mental models, decision state, and trust scales to quantify perceived trustworthiness. Appropriate reliance/trust emerges from aligning actual and perceived trustworthiness and closes the calibration loop toward human-centered and trustworthy AI in healthcare.

Concept and structure. I-HATR organizes human–AI trust research around a user-centric hub, aligning two coordinated pathways. On the XAI (computing) side, the knowledge-production path (model, data, explanation and evaluation) establishes actual trustworthiness. On the HCI (human-factors) side, measures of cognition, affect, decision making, and usability capture perceived trustworthiness. Figure 4 depicts the hub-and-two-wings structure. The left wing asks “Can it be trusted?” and targets objective properties (robustness, fairness, calibration, stability). The right wing asks “Why/how/to what extent is it trusted?” and targets user perception and behavior. Coupled through risk, context, and user constraints, both wings aim for appropriate reliance/trust, avoiding over-trust and under-trust. The framework provides a shared coordinate system for experimental design, metric selection, and interpretation. Section 4.1 and Section 4.2 elaborate the XAI and HCI pathways, respectively.

For positioning, we also relate our framework to Mayer, Davis, and Schoorman’s model of trust decision making [56]. Whereas [56] conceptualizes antecedents of interpersonal trust as Ability, Benevolence, and Integrity (ABI), I-HATR works at the design and evaluation level to align actual and perceived trustworthiness on the same task unit and to enable calibration with joint reporting. Under this mapping, Ability corresponds to model competence, validation, robustness, and calibration; Integrity to data and process governance, transparency, and fairness; and Benevolence to human oversight, safety constraints, and intended-use boundaries. Thus [56] highlights what trust cues matter, while I-HATR specifies how they are operationalized and reported in healthcare AI.

### 4.1. XAI Pathway (Left Wing of the I-HATR)

#### 4.1.1. From Opacity to Explainability: A Task-Oriented Taxonomy for Data and Models

To solve complex problems, many AI systems rely on deep neural networks and other powerful yet opaque mechanisms [95]. This opacity raises concerns about trustworthiness across ethical, technical, and engineering dimensions [96]. A prominent response is to make “black-box” models explain their outputs [87,97,98]. Accordingly, research has shifted toward XAI to increase transparency and interpretability [99]. There is substantial evidence that explanations can support trust formation, which is why XAI is widely viewed as a path to understanding and, potentially, to greater trust—though explanations are not a sufficient condition for appropriate reliance [100].

At the same time, the XAI landscape is fragmented: families of methods, names, and boundaries vary, and evaluation/reporting lacks a common standard [71]. Alignment with human-factors indicators (the framework’s right wing) is also limited. In high-stakes settings such as healthcare, popular post hoc explanations often fail to communicate uncertainty, which can foster over- or under-trust. Guided by our review, we therefore introduce a task-oriented, operational taxonomy under the left wing of I-HATR to support method selection and consistent reporting.

**Figure 5:**
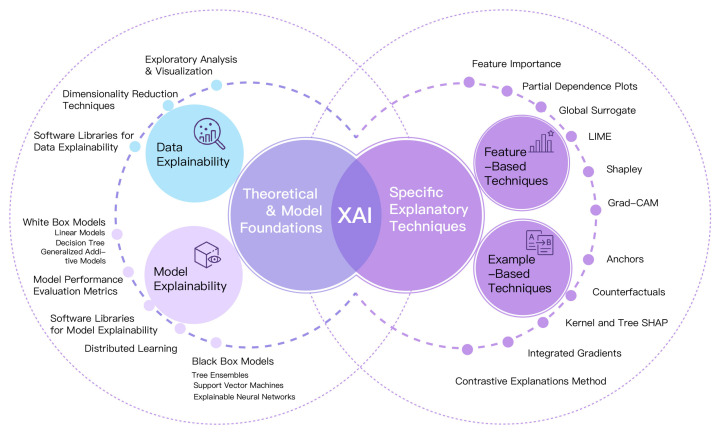
A taxonomy of XAI techniques applicable to data and models.

Figure 5 classifies XAI techniques by target and purpose. Data-centric items (e.g., dataset documentation, slicing, counterfactual data augmentation) are separated from model-centric methods that produce attributions (e.g., SHAP, LIME, Integrated Gradients), saliency/activation maps, examples and prototypes, or rules. The legend indicates typical outputs and the evaluation criteria most relevant to I-HATR: fidelity/stability of explanations, uncertainty quantification, and robustness.

#### 4.1.2. Ante-Hoc (Intrinsic) and Post Hoc Approaches

Building on Kamath and Liu’s three-stage terminology (ante-hoc/intrinsic/post hoc) [101] and Arrieta et al. [87], we summarize two major XAI dimensions in Table 1 and list representative methods, strengths, and limitations with supporting references.

Widely used XAI methods in healthcare. In medical AI, the most prevalent explanation tools are SHAP (SHapley Additive exPlanations), LIME (Local Interpretable Model-agnostic Explanations), and Grad-CAM (Gradient-weighted Class Activation Mapping), all primarily post hoc approaches [87]. They are intuitive and practical, yet they raise concerns about explanation fidelity and stability [115] and often fail to communicate the model’s—and the explanation’s—uncertainty [116]. SHAP has been deployed across hospital readmission prediction, disease-progression modeling, and EHR analytics, contributing to higher perceived transparency and traceability of medical AI [117].

Evidence from imaging and local explanations. For Grad-CAM, Jiang et al. (2020) applied it to diabetic-retinopathy fundus classification, linking heat-map localization of lesions (e.g., microaneurysms) with predicted labels so clinicians could compare model evidence against ground-truth findings, improving perceived transparency and calibrating reliance [118]. However, saliency methods do not always align with true pathology; chest-x-ray benchmarks show limited localization consistency and robustness, which—if unvalidated—can induce inappropriate trust [119]. LIME is also widely used in healthcare. By approximating a complex model’s local decision boundary, it yields case-level explanations; Local Rule-Based Explanations (LORE) complements this by using genetic algorithms to build a synthetic neighborhood for a locally interpretable predictor that outputs rules and counterfactuals, clarifying which factors drive a specific outcome [102,112].

Emerging evidence shows that misleading or unstable explanations can amplify over-trust and degrade clinical decision making [120,121], for example when persuasive but low-fidelity post hoc rationales sway users [122] or when saliency maps fluctuate across near-identical inputs [115]. A further, often overlooked limitation is the sparse communication of uncertainty in both models and explanations [116]. Clinical work requires not only what the model predicts but also how certain it is [123]. User studies indicate that the format of uncertainty communication (frequencies vs. percentages, reference class, visual encodings) materially affects comprehension and reliance; when visualizations do not distinguish confidence from uncertainty, clinicians can be misled—for instance, treating 51% and 95% pneumonia probabilities as effectively “the same” level of evidence [124].

### 4.2. HCI Pathway (Right Wing of the I-HATR)

In computing, explainability methods are a major route to making AI systems more transparent and, in turn, more trustworthy [29,86,87,125]. Recent years have seen a surge of work in this area, yet a widening gap remains between XAI methods and their practical use [92,126,127]. The relationship between explainability and trust is also far from settled; empirical results are mixed and often inconclusive [17,128]. To ensure explainability actually supports appropriate reliance, human-centered evaluation from HCI/HFE is needed to assess both effectiveness and side effects. XAI evaluations tend to emphasize mathematical or algorithmic correctness and faithfulness to the underlying model, whereas HCI/HFE evaluations ask whether, in real tasks, a method produces the intended user effects and improves decisions.

We acknowledge the substantial contributions the XAI community has made to “opening the black box” and improving comprehensibility and trust [98,125]. To bridge the gap between actual and perceived trustworthiness, the I-HATR framework treats the HCI pathway as co-equal with the XAI pathway, not as an add-on. While the left wing (XAI) targets whether a system can be trusted (actual trustworthiness), this section focuses on how people—under task constraints and risk—understand and perceive that trustworthiness. In human–AI interaction, multiple factors jointly shape perceived trust and subsequent reliance, refusal, or abandonment. Based on this view, Section 4.2.1 outlines key determinants of human–AI trust. Consistent with Afroogh et al. [74], we consider person- and context-based influences that generalize across technologies, and we also include factors inherited from trust in automation. Section 4.2.2 then maps the corresponding measurement families (self-report scales, behavioral indicators, and psychophysiology) to technical metrics, supporting study design and cross-disciplinary comparability.

#### 4.2.1. Determinants of Human–AI Trust

Building trustworthiness depends not only on technical levers such as XAI, but also on values and governance. “Black-box” models are generally harder to trust, which is why explainability is a recurring theme [27]. Importantly, perceived trustworthiness often rises with actual trustworthiness, yet it is not determined by accuracy alone; task risk, user mental models, and other moderators also matter. Many studies group determinants into three broad classes—trustor, trustee, and context—though labels and granularity vary across papers [35,74,129]. Following Kaplan et al. within the right wing of I-HATR (the HCI pathway), we organize factors into three categories for clarity: user characteristics, AI-system attributes, and contextual variables [35]. This organization balances theoretical coverage and practical design levers: user factors align with self-report, behavioral, and psychophysiological measures; system factors align with calibration, fidelity/stability, and uncertainty; and context factors capture task risk, workflow, and accountability that shape reliance in healthcare. The triad reduces overlap across constructs and enables joint reporting at the same task unit, improving comparability across XAI and HCI/HFE studies.

- User-related factors

Competence variables such as domain knowledge and technical understanding are generally positively associated with trust [130]. Experience can follow an inverted-U: trust peaks around 2–3 years of use, then declines among >5-year veterans who better appreciate system limits (≈18% drop) [131]. Trait factors also matter: higher innovativeness predicts greater trust, loneliness predicts lower trust, and the effect of extraversion depends on the AI’s form. Demographics (gender, age, SES), general trust propensity, and attitudes toward technology further modulate trust [32,74,132,133,134].

Culture shapes baselines and sensitivities: collectivist settings (e.g., China) emphasize social fit, whereas individualist settings (e.g., the United States) emphasize competence; high uncertainty-avoidance cultures (e.g., Germany) are more error-averse, and German participants have been found to report higher trust than Japanese participants [34]. Personality effects are mixed overall, though openness tends to correlate positively with trust and neuroticism negatively [135]. Negative attitudes on the Negative Attitudes toward Robots Scale (NARS) correlate with lower AI trust across cultures, with East Asian samples scoring ~30% lower negativity than Western samples [136]. Several studies also report higher AI trust among men than women [32,132,133].

- AI-system factors

Compared with traditional automation, algorithmic properties play a stronger role for AI trust. Core levers include performance/accuracy and transparency/explainability [35,137]. Yet performance is not everything: at equal accuracy, how uncertainty and errors are communicated markedly shifts judgments; error patterns affect trust repair—random errors are forgiven and repaired faster than systematic ones [138]. Reliability transparency (e.g., surfacing error rates or confidence intervals) can raise trust more than marginal accuracy gains. Process transparency and explainability exert consistently positive effects, and when systems are adaptive, it is crucial to make operating bounds and state explicit [35,128].

Embodiment and interaction design also shape trust. Physical robots often enjoy higher initial trust due to visibility and anthropomorphic cues; virtual agents sit in the middle; embedded algorithms start lower, reflecting “algorithm aversion” [34,139]. Anthropomorphic cues (voice, tone) can boost initial trust but may inflate unrealistic expectations [34]. Approaching the “uncanny valley” can destabilize trust [140]. Behavior and reputation matter as well: rule compliance, honest disclosure of limits, and consistent behavior support trust acquisition and maintenance, whereas deceptive or unpredictable behavior erodes trust quickly [17].

- Contextual factors

In the AI era, “context” is not a single backdrop but a set of manipulable dimensions. To measure trust (not merely confidence) in studies and practice, designs should (i) expose participants to real or felt consequences (vulnerability), (ii) set initial expectations (how the system is introduced), and (iii) capture attitudes and behaviors with suitable indicators (e.g., reliance/defection/switching). These jointly shape how trust manifests and evolves [30]. Large-scale evidence further elevates “shared context” to a third pillar alongside trustor and trustee, supporting a dimensionalized view of context [35,141]. Operable dimensions include task domain and risk (stakes and uncertainty), time/interaction history (dynamic trust), and social/organizational setting (roles and group cues).

By task domain and risk, “who is more trusted” is domain-specific: users more readily defer to robots/algorithms on functional or technical tasks, rely more on humans for socio-emotional tasks, and the gap narrows on hybrid tasks [142]. Risk shifts not only overall trust levels but also the slope from trust to reliance: under high stakes, users scrutinize errors and boundaries more, so design should target appropriate reliance [31].

Over time and across social/organizational settings, trust is plastic—gains, losses, and repair occur dynamically. Real-time successes and failures update trust immediately [143]; asymmetric adjustment is observed as reliability trends up vs. down [144]. Contextualized explanations and “when not to use” boundary cues can slow deterioration after surprises or violations [145]. In groups, framing the AI as teammate vs. opponent sets different baselines, and observational or social contagion can transmit others’ trust to bystanders [33,146].

#### 4.2.2. Evaluation and Measurement of Human–AI Trust

Beginning with the automation era, research has advanced along two intertwined tracks: what shapes trust, and how to measure it. In the human–AI collaboration era, measurement has moved beyond single indicators toward three complementary families—self-report, behavioral, and psycho-/neurophysiological—often combined in multimodal, time-resolved designs (e.g., continuous sampling during embodied interaction) to capture how trust fluctuates with task and time [30].

- Self-report measures

Questionnaires capture users’ subjective assessments of a system’s ability, reliability, and benevolence [62]. Kohn et al. review 16 self-report approaches spanning generic short forms and scenario-specific instruments; widely used tools include the Trust in Automation (TiA) scale [62], the TPS-HRI for human–robot interaction (Schaefer, 2016) [147], and Human–Computer Trust (HCT) scale [148]. Strengths are efficiency and construct coverage; limitations include recall bias and imperfect correspondence to actual reliance, underscoring the need to pair self-report with behavior.

- Behavioral measures

Behavioral indices operationalize whether users actually rely on or delegate to the system: reliance/compliance rate, joint performance, agreement rate, response latency, and task-specific paradigms (e.g., “trust-fall” style tests), as well as observations of over-trust in evacuation or alarm scenarios, and decision tasks that manipulate false-alarm/miss trade-offs to quantify dependence [149,150,151,152]. These measures align closely with safety and workflow outcomes but require careful control of risk, incentives, and feedback loops.

- Psycho-/neurophysiological measures

Objective signals—electrodermal activity (EDA), heart rate/HRV, EMG, eye-tracking, EEG, and fNIRS—enable low-intrusion, continuous tracking of arousal, cognitive load, vigilance, and attentional allocation. When triangulated with self-report and behavior, they support convergent validity in high-risk settings from clinical AI to real-world driving [82,153,154,155,156,157]. In practice, studies often combine one or more modalities and analyze alignment both across types (e.g., trust ratings vs. reliance) and within type (e.g., multiple behavioral proxies) [147,158].

To strengthen inference, pair subjective trust scales with task-embedded behavioral measures and at least one low-burden physiological channel (e.g., eye-tracking or HRV). Time-lock all data streams to salient events (alerts, model errors, human overrides). Report not only trust levels but also their dynamics—growth, breakdown, and repair—under explicitly described task risk, time pressure, and accountability conditions. This improves comparability, reproducibility, and relevance to real-world deployment.

## 5. Discussion

### 5.1. Paradigm Shifts and the Evolution of Measurement

This narrative review offers a critical synthesis of roughly three decades of research on human–technology trust. The evidence shows a shift from automation-era questions that centered on reliability and predictability (“can it be trusted?”) to AI-era concerns about appropriate reliance (“how should we rely on it?”). In parallel, trust measurement has expanded from single self-report scales to combined portfolios of self-report, behavioral, and physiological/neural indicators [147,154]. Given the limits of human cognition and current AI methods, there is still no consensus on how to quantify an optimal level of clinician–AI trust that yields the most accurate and dependable clinical decisions. Consistent with prior observations, questionnaire-based measures remain dominant in medical AI, but the use of behavioral indicators is increasing [30,159]. On the physiological side, eye-tracking in radiology is comparatively mature and can capture visual search load and decision processes, which indirectly map the dynamics of trust and reliance. With respect to ecological validity, workflow-integrated deployments—such as presenting “model facts labels,” constraining to well-specified use cases, and surfacing boundaries of applicability—help translate actual trustworthiness into perceived trustworthiness and reduce misuse or overreach [78,130].

Most studies can be organized within a tripartite “user–system–context” frame. However, outside automated driving, the prevailing evidence still relies on short-duration, low-risk experiments; reports on external validity and reproducibility in high-risk clinical settings remain limited, which constrains cross-context generalization. Moreover, “trust” is frequently conflated with acceptance, usefulness, or satisfaction, and subjective trust does not always align with actual reliance or delegation, limiting cross-disciplinary comparability.

### 5.2. From Parallel Tracks to Resonance: Aligning XAI and HCI

Explanation and uncertainty communication have become central levers for fostering understanding and appropriate reliance. Popular post hoc methods (e.g., SHAP, LIME, Grad-CAM) are intuitive, yet joint reporting of explanation fidelity/stability and uncertainty remains uncommon, especially in high-risk tasks, which weakens the actionability of findings. We also observe a gap between XAI-side metrics (e.g., calibration, explanation fidelity) and HCI/HFE-side trust outcomes (e.g., reliance/overreliance/underreliance, trust calibration): parallel reporting and a minimal alignment set are often missing, making evidence hard to accumulate across studies. In our I-HATR framework, we place both tracks in one coordinate system: the user sits at the center; the XAI “left wing” shapes actual trustworthiness through model–data–explanation–evaluation; the HCI/HFE “right wing” characterizes perceived trustworthiness under concrete user–task–context constraints. The goal is calibration toward appropriate trust/reliance. The framework does not subsume HCI under XAI or substitute perceived trust for explainability; instead, it provides an iterative evidence channel that links methods to measures and supports human-centered and trustworthy AI by design.

Practically, we recommend reporting calibration, explanation fidelity, and stability on the XAI side, while simultaneously observing on the HCI/HFE side: subjective trust, reliance, overuse, disuse, abandonment, and cognitive load/conflict. This preserves technical reproducibility and directly maps human-factor measures to decision outcomes, reducing the risk—documented in high-stakes settings—of “strong technical scores but user-side mismatch” [17,25,92].

### 5.3. Operationalizing Complexity

We are entering the AI era in earnest. Analogous to how “horsepower” made steam power tangible, AI needs stakeholder-facing representations of trust. Trust is a subjective psychological state, whereas reliability is an objective probabilistic property; they should not be conflated. Researchers should use the terms explainability, interpretability/understandability, and transparency in a disciplined and consistent manner. We advocate tiered disclosure: in high-risk domains such as healthcare, require co-presentation of uncertainty, calibration, and traceability; in lower-risk domains, use lighter information burdens. Also distinguish trustworthiness/reliability (objective) from trust (subjective): high reliability does not guarantee trust, and convincing demonstrations can inadvertently induce overtrust and overreach. Fairness affects both objective trustworthiness and perceived trust; explanation and transparency can bridge the two. Finally, trust is bidirectional: not only should people trust AI appropriately, but AI systems should constrain when and how they “trust” humans through role-appropriate permissions, abuse safeguards, and compliant disclosure controls. By jointly calibrating user reliance and system disclosure, we can approach a steady state between safety and usability.

### 5.4. Compliance and Societal Implications

This manuscript centers on theory and method. To support practical use in healthcare settings, we add a concise discussion of compliance and societal implications aligned with the proposed framework. For high-risk applications, studies and reports should include external validation, robustness testing, and descriptions of failure modes, together with clear statements of intended use and typical failure domains. Joint reporting of calibration outcomes and uncertainty ranges for the same task helps define interpretive boundaries, improves decision transparency, and supports appropriate reliance. Human oversight requires explicit review points, escalation pathways, role responsibilities, and the ability to interrupt or override system outputs. Data governance and traceability call for documenting data subsets, model versions, and system logs to enable audit and reproducibility. Implementation further carries technological, economic, social, ethical, and legal implications, including deployment costs, equitable resource allocation, societal acceptance, privacy and fairness risks, and potential legal liability and regulatory fit. These anchors are not a jurisdiction-specific legal checklist but pragmatic cues that can be adapted in concrete projects.

## 6. Limitations, Research Gaps, and Future Directions

### 6.1. Limitations

This study is a critical narrative review that includes only peer-reviewed, English-language publications (1995–June 2025). We used a purposeful, iterative search strategy rather than an exhaustive systematic review, and we did not perform a formal risk-of-bias assessment or meta-analysis. As a result, database, language, and selection biases may be present. Given the English-only, peer-reviewed corpus and the search window (1995–June 2025), coverage is skewed toward Western, high-income health systems and excludes gray literature; combined with the absence of formal risk-of-bias scoring, this likely amplifies publication bias and limits transferability to other jurisdictions.

We situate trust in medical AI within the broader literature on human–machine trust. A substantial portion of the included evidence comes from short-duration, low-risk, laboratory settings with non-specialist samples, which limits external validity and transportability to high-stakes healthcare environments. In addition, some studies appear to conflate the constructs of “trust/trustworthiness” with “perceived usefulness/satisfaction.” Reporting of behavioral outcomes (e.g., reliance, disuse, misuse) and key contextual variables (e.g., risk, time pressure, accountability structures) is often incomplete, which weakens comparability across studies. Two additional constraints warrant note. First, our task-level crosswalk is conceptual and cannot reconcile inconsistent metrics quantitatively; it is better understood as design scaffolding rather than a prescriptive checklist. Second, rapid model and data versioning means findings tied to specific releases may drift; our search closed in June 2025, and we did not re-audit versions thereafter.

Finally, while our I-HATR framework proposes a hub-and-two-wings juxtaposition to align XAI and HCI/HFE, we do not claim a one-to-one mapping at the level of every metric or procedure. The correspondences offered are intended to be illustrative and generative rather than exhaustive.

### 6.2. Research Gaps and Future Directions

Scientific progress invariably introduces new challenges and opportunities; research on human–machine trust (automation and AI) is no exception. Below we outline several gaps and corresponding opportunities that are likely to shape the agenda in the coming years.

(1) Bridge the split between system metrics and human measures.

To improve comparability and reproducibility, future studies should co-report two evidence chains within the same experiment. Technical/XAI side: calibration, faithfulness/stability, and uncertainty; HCI/HFE side: trust scales, perceived trust, cognitive measures, and psychophysiology. Studies should also annotate task risk, time pressure, and accountability structures; disclose model versioning, data slices, effect sizes, and confidence intervals; and supply reusable figure templates and reporting checklists.

Rationale. XAI evaluations often emphasize model-side competence (robustness, fidelity, calibration), while HCI/HFE studies emphasize user-side outcomes (trust, reliance/over- or under-use, cognitive load); evidence rarely accumulates on the same task unit; Opportunity. Pair technical metrics with human-factors measures in a single task to enable calibration and interpretation; What to report. At minimum, report calibration, fidelity/stability of explanations, and robustness to salient perturbations, alongside validated trust scales, reliance behavior (over-/under-reliance rates), and cognitive load (e.g., brief NASA-TLX or a psychophysiological proxy), plus task risk and time pressure.

(2) Close the “performance–deployability” gap in healthcare.

Many reports show that, for specific diagnoses or treatment selection, medical AI often outperforms clinicians [80,160]. Yet these gains typically arise on controlled datasets and offline evaluations; limited explainability and poor uncertainty communication hinder appropriate trust and real-world adoption. Technical fixes from XAI alone cannot resolve the “explainable ≠ trustworthy” mismatch. We therefore call for public benchmark tasks and datasets that jointly cover explanation quality, uncertainty quantification, and trust calibration, along with cross-disciplinary metrics and reporting checklists to align XAI and HCI evidence. Measurement should also expand beyond questionnaires to include behavioral and psychophysiological signals, plus outcome metrics—efficiency/effectiveness, safety/privacy/control, output credibility, and advice reliance—and an explicit appraisal of system vulnerabilities and risks.

Rationale. High offline performance does not guarantee safe, sustainable deployment in clinical workflows; Opportunity. Tie evaluation to intended use and risk class, including workflow integration and error recovery paths; What to report. External validation, out-of-distribution checks, failure modes with representative cases, human-in-the-loop escalation paths, and any changes in task allocation or accountability.

(3) Ethics, culture, and time horizons.

Bias, privacy, and fairness materially shape trust and require dedicated ethical evaluation frameworks [81]. Current theory is heavily Western-centric; for example, 92% of samples in trust studies come from Western cultures, with <1% from the Middle East, Africa, or Latin America [34], limiting generalizability. Recent work highlights the need for cross-cultural experiments and long-term tracking [161]. Most dynamic-trust studies focus on short sessions (hours), whereas real deployments unfold over years. While deployments may span multiple years, we do not attempt forecasts beyond two years; feasibility should be treated as conditional and revisited at planned intervals. Meta-analytic evidence suggests longitudinal studies rarely exceed six months [33]. This mismatch obscures phenomena such as seasonal “trust fatigue.” Recommendations:

- Conduct real-user longitudinal studies over 12–24 months with scheduled interim assessments; extensions beyond this window should be justified by interim findings;
- Examine generational differences (e.g., Gen Z vs. Millennials) in AI trust;
- Develop synchronous trust–capability assessment models to detect asynchrony in real time;
- Apply complex-systems approaches (e.g., differential-equation models) to capture nonlinear trust dynamics and “butterfly effects.”

(4) Rebalance underexplored areas.

The literature skews toward one-way “human trusts AI,” with limited attention to “AI’s trust in humans” or “AI–AI trust.” Work clusters around explainability, trust factors, and measurement, while empathy, privacy, fairness, accountability, and novel metrics remain thin. We recommend prioritizing high-stakes scenarios, building cross-disciplinary indicator sets, systematically filling these low-density cells, and validating their impact on reliance and safety.

Specifically, in high-stakes clinical tasks (e.g., diagnosis, triage, medication safety), underexplored areas can be advanced by designing dyadic or triadic protocols that observe not only human trust in AI but also AI’s trust in human operators (e.g., abstention, querying, or escalation conditioned on operator competence and context) and trust between AI systems (e.g., detection and arbitration of cross-model disagreement). To make evidence commensurable, studies should pair technical signals—calibration, uncertainty, out-of-distribution checks, explanation fidelity and stability, abstention rates, and inter-model disagreement—with human-side outcomes—validated trust scales, reliance and override behavior, brief empathy/communication measures, and cognitive-load indicators—and organizational markers such as oversight roles, escalation latency, and accountability mapping. Reporting should specify the task and risk class, intended-use boundaries, privacy safeguards, and subgroup-wise performance together with calibration and fairness diagnostics and any mitigation. Where feasible, align measures longitudinally on the same task unit within a 12–24-month horizon with pre-specified interim assessments; model seasonality and workload to detect trust fatigue; document retention and missing-data handling; and preregister hypotheses and analysis plans. Finally, share reusable artifacts (e.g., prompts, interface screenshots, logging schemas) and include negative or null results to enable cumulative evidence and translation across settings.

(5) From one-way trust to reciprocal trust.

As next-generation AI becomes more autonomous, learns continuously, and invokes external tools, trust should evolve from unilateral (“humans trust AI”) to reciprocal human–AI trust. On the human side, explanation and uncertainty communication should support appropriate reliance; on the machine side, controlled disclosure and permission gating should yield auditably trustworthy-to-humans behavior (e.g., intent/qualification checks). Concretely, machine-side trust signals should be made explicit: estimate operator competence and protocol adherence from recent behavior and context, set confidence and risk thresholds for abstention or co-signature, throttle external-tool invocation under uncertainty, and log each trust decision as a first-class event. We urge academia and industry to adopt bidirectional calibration as a common goal: tailor disclosure tiers and revocable authorizations to task risk and role responsibility, and build a traceable evidence chain—in healthcare, for example: patient-level uncertainty and confidence intervals, model versions and data slices, use boundaries and decommission rules, AI intervention points and order-change audit trails, linkage to clinical outcomes and safety events. Complement this with longer-horizon HITL evaluations to test whether mutual trust actually reduces misuse/overuse and strengthens collaboration. Before full autonomy arrives, embedding these mechanisms into research and governance baselines will help achieve a demonstrable steady state between safety and usability and lay guardrails for reliable deployment.

(6) Regulatory adaptability.

Finally, future work should examine regulatory adaptability, that is, how trust metrics and reporting templates can be aligned with evolving guidelines across jurisdictions and how cross-national differences affect deployment and calibration in healthcare. Studies should test whether joint reporting of calibration and uncertainty meets both scientific transparency and compliance documentation needs. We also encourage protocol development co-designed with regulators and clinical partners to ensure evaluability in real settings.

## 7. Conclusions

This review traced three decades of work as the field shifted from trust in automation to trust in AI, detailing changes in paradigms, methods, and focal questions. To close the gap between the computer science pathway (XAI) and the human factors/HCI pathway, we proposed the I-HATR framework as a shared grammar that aligns evidence, measures, and limitations across both tracks. Although we ground it in healthcare, the framework generalizes to broader AI trust settings. Building on this two-track view, we analyzed data-and-model issues in XAI, key determinants of human–AI trust, and multi-level evaluation methods, and we mapped actual trustworthiness to perceived trustworthiness within a single coordinate system to support human-centered and trustworthy AI in healthcare.

Looking ahead, we recommend co-reporting explanation quality, uncertainty, calibration, and user perceptions within the same study while making task and organizational context explicit, to enable comparable, transferable, and reproducible evidence. In healthcare, this means embedding intended use, off-label boundaries, patient-level uncertainty displays, trust calibration, and selective HITL evaluations into the workflow for high-stakes tasks such as CDSS, imaging, and triage. The goal is not to “maximize trust” but to achieve appropriate reliance. The I-HATR framework offers an actionable path for reusable evaluation and responsible human–AI system design, and it lays a scalable foundation for future movement toward reciprocal human–AI trust and governance. We close with a call for sustained collaboration across XAI and HCI/HFE, uniting engineering, computer science, the social and behavioral sciences, psychology and neuroscience, ethics, and law to address the remaining challenges and advance human-centered, trustworthy medical AI and, more generally, trustworthy AI in other domains.

## Figures and Tables

**Table 1:** Comparison of post hoc and ante-hoc XAI approaches.

Dimension	Post Hoc Explanation [102]	Ante-Hoc Explanation [19]
Definition	Explains a trained model’s decisions after training and prediction via external tools [103].	Builds explainability into the model during design/training so predictions and explanations co-emerge; the decision process is inherently understandable [22].
Representative models/methods	SHAP [22] LIME [104] CAM/Grad-CAM [105] LORE [106]	Decision trees [107]; linear/logistic regression [108]; rule-based models [109]; GAM [110]; interpretable neural networks [111].
Strengths	Model-agnostic and flexible; can explain complex black-box models [112].	Explanations are faithful to model behavior without extra approximations; typically more reliable [22].
Limitations	Explanations are often approximations that may diverge from the true decision logic [113].	Expressive power can be limited; may trade off some predictive performance [114].

## Data Availability

Derived data supporting the findings of this study are available from the authors upon request.

## Supplementary Materials

The following supporting information can be downloaded at: https://www.mdpi.com/article/10.3390/bioengineering12101070/s1, Table S1: Representative keywords and database-specific search terms.
